# Self-Reported Post-COVID Symptoms at 18 Months After Infection Among Adults in Southern Brazil: A Cross-Sectional Study

**DOI:** 10.3390/healthcare13030228

**Published:** 2025-01-23

**Authors:** Franciele Aline Machado de Brito, Carlos Laranjeira, Marcia Moroskoski, Maria Aparecida Salci, Stéfane Lele Rossoni, Wanessa Cristina Baccon, Rosana Rosseto de Oliveira, Priscila Garcia Marques, Herbert Leopoldo de Freitas Góes, Fernanda Fontes Mello, Flávia Renata Baldissera da Cruz Blaszczak, João Ricardo Nickenig Vissoci, Jesús Puente Alcaraz, Luiz Augusto Facchini, Lígia Carreira

**Affiliations:** 1 Department of Postgraduate Nursing, State University of Maringá, Paraná 87020-900, Brazil; pg54936@uem.br (F.A.M.d.B.); mmoroskoski@uem.br (M.M.); masalci@uem.br (M.A.S.); wanessa.baccon@gmail.com (W.C.B.); prof.rosanarosseto@uninga.edu.br (R.R.d.O.); hlfgoes@uem.br (H.L.d.F.G.); lcarreira@uem.br (L.C.); 2 School of Health Sciences, Polytechnic University of Leiria, Campus 2, Apartado 4137, 2411-901 Leiria, Portugal; 3 Centre for Innovative Care and Health Technology (ciTechCare), Polytechnic University of Leiria, Campus 5, 2414-016 Leiria, Portugal; 4 Comprehensive Health Research Centre (CHRC), University of Évora, 7000-801 Évora, Portugal; 5 Postgraduate Department of Health Sciences, State University of Maringá, Paraná 87020-900, Brazil; pg908463@uem.br; 6 Department of Physical Education, State University of Maringá, Paraná 87020-900, Brazil; pgmarques@uem.br; 7 Department of Undergraduate Nursing, State University of Maringá, Paraná 87020-900, Brazil; ra81374@uem.br (F.F.M.); pg405951@uem.br (F.R.B.d.C.B.); 8 Division of Emergency Medicine, Duke Global Health Institute, Duke University, Durham, NC 27708, USA; jnv4@duke.edu; 9 Department of Nursing, University of Burgos, 09001 Burgos, Spain; jpalcaraz@ubu.es; 10 Department of Social Medicine, Postgraduate Programs in Epidemiology, Nursing and Family Health, Federal University of Pelotas, Rio Grande do Sul 96010-610, Brazil; luizfacchini@gmail.com

**Keywords:** SARS-CoV-2, long COVID, signs and symptoms, cross-sectional, Brazil

## Abstract

Background/Objectives: Currently, there is a limited understanding of the long-term consequences following acute COVID-19, referred to as long COVID. This cross-sectional study aims to analyze the prevalence of persistent signs and symptoms of long COVID, 18 months after primary SARS-CoV-2 infection in adults in southern Brazil. Methods: Using two national databases (the digital registry of SARS-CoV-2 positive cases), 370 individuals living in the state of Paraná (Brazil) were recruited. Data were collected through telephone interviews conducted in 2021 and 2022. Results: The overall prevalence of long COVID was 66.2% among study participants. During the acute phase of infection, the most common symptom clusters included neurological symptoms (87.0%; n = 318), followed by respiratory (82.0%; n = 301), musculoskeletal (66.0%; n = 241), digestive (50.0%; n = 184), psychological (38.0%; n = 138), and endocrine symptoms (28.0%; n = 104). In the 18 month follow-up, the main persistent symptoms were memory loss (42.7%), fatigue (32.2%), anxiety (23.5%), dyspnea (19.7%), and hair loss (19.7%). The proportion of participants with long COVID was statistically higher in females (73.9%), those with a family income below two minimum wages (94.7%), those who do not practice physical activity (83.3%), those who report poor sleep quality (93.3%), those who use long-term medication (85.9%), those who needed health care in the previous six months (87.3%), those who required professional and/or family care (79.3%), those who were in the ICU (79.0%), and those who used ventilatory support (77.5%). Conclusions: Long COVID is a complex condition that requires long-term monitoring and investment in health services due to its high prevalence and the health consequences in the population.

## 1. Introduction

In May 2023, the World Health Organization (WHO) declared the end of the emergency phase of COVID-19. By December of the same year, more than 772 million cases and almost seven million deaths had been reported worldwide [1]. In Brazil, the second country with the highest number of deaths, 38,867,008 cases and 712,957 deaths were recorded in August 2024. The state of Paraná, located in the southern region of the country, was responsible for 7.8% of all confirmed cases, resulting in 46,951 deaths [2].

During the pandemic, most people infected with SARS-CoV-2 recovered fully. However, the WHO states that a significant percentage of individuals develop a diversity of symptoms in the medium and long term, known in the literature as acute post-COVID-19 syndrome or long COVID [3]. Long COVID is an umbrella term for what “occurs in people with a history of probable or confirmed SARS-CoV-2 infection and is characterized by the presence of signs and symptoms three months after the primary coronavirus infection, which remain for at least two months, which are not explained by alternative diagnoses” [3]. The current clinical definitions of long COVID proposed by WHO (World Health Organization) mention that persistent symptoms have a duration of at least 2 months, while other organizations like the CDC (Center for Disease Control and Prevention), and the NICE (National Institute for Health and Care Excellence) indicate at least 3 months. More recently, in 2024 NASEM (National Academies of Sciences, Engineering and Medicine) defined long COVID as “an infection-associated chronic condition that occurs after SARS-CoV-2 infection and is present for at least 3 months as a continuous, relapsing and remitting, or progressive disease state that affects one or more organ systems” [4]. The challenge in formulating an integrated definition of long COVID extends to its diagnosis, suggesting a risk of underdiagnosis of the condition [5]. The origin of persistent symptoms is still being investigated; however, preliminary research points to exacerbated immune reactions, previous comorbidities, severity of the disease in the acute phase, and even emotional factors [6,7,8]. Hence, current diagnostic and treatment options are insufficient, necessitating numerous clinical studies to thoroughly evaluate interventions targeting proposed underlying biological pathways, such as viral persistence, neuroinflammation, excessive blood coagulation, and autoimmunity [9].

The real prevalence of long COVID is unknown [10], because there is no information system with these records. More precisely, its estimated rate is 10–30% of non-hospitalised cases, 50–80% of hospitalised cases, and from 10% to 12% of vaccinated populations [10,11,12]. While more robust evidence is needed to determine the effectiveness of vaccines in preventing and treating long COVID, a recent review suggests that COVID-19 vaccines might have protective and therapeutic effects with long COVID [13]. However, these estimates fluctuate based on the study design, including factors such as the study population, definition of post-COVID circumstances, data source, follow-up duration, time period, and main variant [11].

In a Brazilian cohort of 1822 laboratory-confirmed SARS-CoV-2 infections, the global prevalence after 12 months was 64.2%. Prevalence varied across three levels of exposure to COVID-19’s acute phase: ambulatory (31%), medical ward (31%), and intensive care unit (38%) [14]. As for the prevalence of long COVID and the multiplicity of symptoms presented, the studies vary widely according to the characteristics of the population and the follow-up time. However, there is consensus that this is a systemic condition that affects multiple organ systems with different impacts, causing a multitude of symptoms [13]. Among the wide range of symptoms (more than 200), the most common symptoms are fatigue [15,16], headache, dyspnoea, memory loss, smell and taste disorders, chest pain, and changes in sleep quality [6,7,17].

There is a lack of information on long-term clinical signs and symptoms that may contribute to the occurrence of this condition. Research reveals that long COVID interferes with quality of life, functional dependence, work capacity, and social relations, causing physical and emotional suffering [18,19,20,21,22]. Long COVID survivors experience changing symptoms, leading them to independently manage their health conditions and feel uncertain about whether to tolerate these symptoms or seek medical attention. This attitude indicates a lack of trust in the capacity of healthcare services to address their needs after the COVID-19 pandemic [21]. Moreover, based on scientific data, many public health resources are still needed to cope with this condition. As few studies extended their surveillance of long COVID after 12 months, this study sought to analyze the prevalence of persistent signs and symptoms 18 months after primary SARS-CoV-2 infection in adults from southern Brazil.

## 2. Methods and Materials

### 2.1. Study Design

This is a cross-sectional study developed by the COVID-19 Cohort Research Network in the state of Paraná, in partnership with the State University of Maringá (UEM), Paraná State Health Department (SESA), the Federal University of Pelotas, and Duke University [23]. The recommendations of the Checklist Strengthening the Reporting of Observational Studies in Epidemiology (STROBE) were used as guide in reporting the study findings [24].

### 2.2. Setting and Recruitment

The study site was the state of Paraná, located in the southern region of Brazil, with a population of 11,444,380 inhabitants, according to the 2022 demographic census, and a Human Development Index of 0.769 in 2021, considered high human development [25].

Participants were selected from two databases: the Influenza Syndrome Epidemiological Surveillance System (SIVEP-Gripe) and the Notifica COVID-19 Paraná. Notifica COVID-19 Paraná is a database implemented by SESA, which communicates with the Central Laboratory of the State of Paraná (LACEN/PR), where all cases confirmed by laboratory examinations are registered [26]. Consultation of both databases provided greater coverage of COVID-19 cases in the state, from the milder cases that tested positive but did not require hospitalization (registered in the Notifica COVID-19 Paraná database) to those hospitalized with severe acute respiratory syndrome (registered in SIVEP-Gripe). Records found in both databases were excluded after careful analysis, comparing date of birth, full name, and parents’ names, with remaining information from SIVEP-Gripe.

### 2.3. Sample Size and Participant Eligibility

The sample size was calculated using the single-population-proportion formula with a precision corresponding to an effect size of 5% and assuming a prevalence of long COVID of 30% two-years after COVID-19 infection [16]. There is substantial heterogeneity in prevalence estimates due to differences in population characteristics (hospitalized vs. non-hospitalized) and methodology [16]. Hence, accounting for a 20% allowable error and a 20% loss to follow-up, the sample size was calculated to be 360.

Study participants were included in the study if they were adults (aged from 18 to 59), living in the state of Paraná, with a confirmed COVID-19 diagnosis through the reverse transcription test (RT-PCR), between March and December 2020. Pregnant women and those who died were excluded. The diagnosis period encompasses individuals whose symptoms began prior to 7 June 2021, at which point over 75% of patients were infected with the Alpha variant [27].

Initially, 695 people were selected. After applying the eligibility criteria, 324 were excluded because they were older people (≥60 years old) and one individual had missing data in the database, resulting in 370 participants.

### 2.4. Data Collection

Data collection occurred 18 months after infection and was performed by nurses and undergraduate and graduate students, after training in “relational” skills (40 training hours) such as understanding the content, ensuring consistent responses, and minimizing biases during data collection. Training in interview facilitation skills also encompassed building trust and rapport, active listening, employing a nonjudgmental stance, and reducing interviewer bias. The interviews, lasting from 40 to 60 min, took place between September 2021 and June 2022. They investigated the persistence of COVID-19 signs and symptoms. Participants did not receive remuneration for their participation. All questions were standardized using an interview guide to ensure consistency among interviewers. The COVID-19 Paraná/UEM Cohort researchers and the partner institutions prepared and validated the data collection form [23].

The first form of contact was by telephone in which people were invited to participate in the research, the informed consent form (ICF) was read, and if accepted, the ICF was sent to the participant by mail, email, or WhatsApp^®^ (version: 2.3000.1015010030), according to preference. When the individual could not grant the interview at the time of contact, an appointment was made for the day and time of their choice.

### 2.5. Study Variables

The independent variables were sex [male/female]; age group [18 to 30, 31 to 45, 46 to 59]; race/ethnicity [white or non-white]; years of education [<8 years of study or ≥8 years of study]; married or with partner [no/yes]; lives alone [no/yes]; family income [<2 minimum wages or ≥2 minimum wages]; smoking habit [no/yes]; physical activity practice [“Currently, do you practice any type of physical exercise or sport?”, with the response options (1) no, and (2) yes]; quality of sleep pattern [“Currently, how do you rate the quality of your sleep?”, with the response options (1) bad, and (2) good]; presence of comorbidity [no/yes]; long-term medication use [no/yes]; need of social or family support [no/yes]; place of treatment of COVID-19 [ambulatory/inpatient ward/intensive care unit]; use of ventilatory support [no/yes]; need of healthcare support in the last six months [no/yes]; used the Unified Health System [no/yes] (Unified Health System is the Brazilian health program that guarantees full, universal, and free access for the entire population of the country); and the presence of persistent symptoms in the main organ systems [neurological/respiratory/digestive/endocrine/skin/skeletal/circulatory and psychological symptoms].

The dependent variable was the presence of long COVID, following the definition proposed by NICE (National Institute for Health and Care Excellence), that is the presence of symptoms three months after disease onset that are not explained by alternative diagnoses [28]. 

### 2.6. Data Analysis

Descriptive statistics were reported as means with standard deviation (S.D.) or medians with inter-quartile range (IQR) for continuous variables, while categorical variables were denoted as frequencies and percentages, contingent on normality distribution, as verified by the Anderson-Darling test. Sociodemographic and health characteristics between individuals with long-COVID and those without long-COVID were compared. Wilcoxon rank sum tests were used for continuous variables, and Chi-square or Fisher’s exact tests were used for discrete variables, as appropriate. *p*-values  <  0.05 indicated statistical significance. All statistical analyses were performed in the software R (R Core Team 2023).

### 2.7. Ethical Issues

The study was approved by the Permanent Committee on Ethics in Research with Human Beings of UEM, under the opinion number: 4165272 and CAAE: 34787020.0.0000.0104 on 21 July 2020. For the data extracted from SESA, the authorization was granted by Worker’s Hospital, responsible for responding to the research requests of this secretariat, under the opinion number: 4214589 and CAAE: 34787020.0.3001.5225 on 15 August 2020.

## 3. Results

### 3.1. Sociodemographic and Health Characteristics of Sample

In total, 370 patients were analyzed, of which 245 developed long COVID (reported at least one symptom continuing) and 125 manifested symptoms only in the acute phase, that is, in the first 14 days after the first symptom.

The prevalence of long COVID was 66.2% among infected individuals. Table 1 shows the absolute and relative frequencies of the study variables in their different levels, as well as the distribution of groups with or without long COVID.

Regarding sex, the proportion of individuals was well balanced at 56.49% male and 43.51% female. As for the age group, 16.22% were between 18 and 30, 36.76% between 31 and 45, and most participants were aged between 45 and 59 years (47.02%). The race/ethnicity of most participants was white (42.16%), while 18.65% were non-white. In terms of schooling, 51.62% had more than eight years of schooling and only 7.57% had less than eight years of schooling. Most had a partner (55.14%) and only 8.38% lived alone. Nearly 41% had a family income greater than two minimum wages. In relation to life habits, 17.03% were smokers, 59.46% practiced physical activity, and 64.87% reported sleep quality as good.

Regarding health conditions, almost half of the sample (44.86%) had a comorbidity and 21.08% used long-term medication. Around 38% needed health care services in the last six months. Among the participants, 23.51% needed professional and/or family care during their recovery from COVID-19. Relating to the place of treatment in the acute phase of the disease, 36.76% were ambulatory, 31.08% in wards, and 32.16% in the intensive care unit (ICU). Concerning treatment, 43.24% used some type of ventilatory support and 40.54% used the health services of the Unified Health System.

The proportion of those with long COVID was statistically higher in females (73.91%), in individuals with family income below two minimum wages (94.74%), in those who do not practice physical activity (83.33%), in those who report poor sleep quality (93.33%), in those who use long-term medication (85.90%), in those who needed health care in the last six months (87.32%), in those who required professional and/or family care (79.31%), in those who underwent ICU (78.99%), and also those who used ventilatory support (77.50%).

### 3.2. Course of Symptoms over Time Among Participants

**Figure 1:**
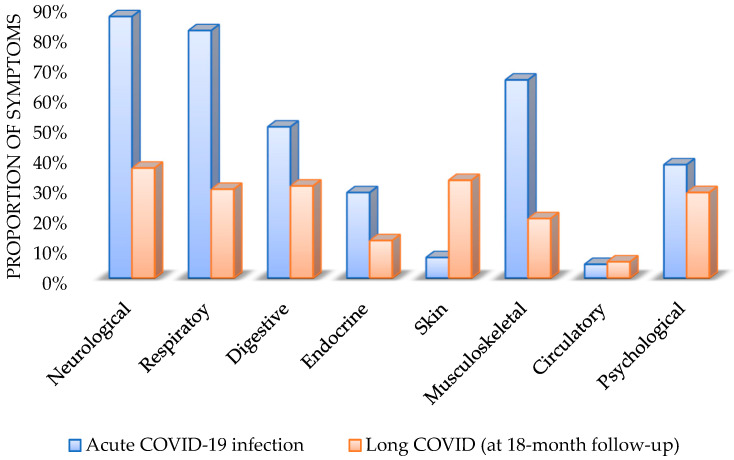
Evolution by symptom clusters: acute COVID infection and long COVID (at the 18 month follow-up).

Table 2 and Figure 1 show the prevalence of symptoms in each organ system, referring to acute COVID-19 infection and long COVID (at the 18 month follow-up). Due to the multiplicity of signs and symptoms, these can be viewed in Supplementary Table S2; however, they are described in this section. Over time, most of the symptom clusters became less frequent, except skin and circulatory symptoms which increased at the 18 month follow-up.

During the acute phase of infection, the most common symptom clusters included neurological symptoms (87%; n = 318), followed by respiratory (82%; n = 301), musculoskeletal (66%; n = 241), digestive (50%; n = 184), psychological (38%; n = 138), and endocrine symptoms (28%; n = 104).

At the 18 month follow-up: 36.49% of the participants had at least one neurological symptom, such as a headache (13.51%); change in vision (18.92%); change in smell (9.19%); change in taste (6.49%); change in speech (6.49%); tinnitus in the ear (8.11%); tingling or numbness in any part of the body (12.70%); dizziness (9.46%); loss of coordination of movements (10.54%); and memory loss (42.70%).

Regarding respiratory symptoms, 29.46% of the sample showed at least some alteration, such as a runny nose (6.49%); sore throat (9.73%); hoarse voice (8.38%); cough (10.81%); production of phlegm (7.84%); chest pain (9.19%); and dyspnoea (19%).

Among the symptoms related to the digestive system, 30.54% reported at least some disorder, such as a change in appetite (10.27%); nausea (4.32%); vomiting (2.43%); abdominal cramps/pain (4.59%); and change in stool (4.86%).

In relation to endocrine symptoms, 12.43% of respondents had at least one symptom, such as hair loss (19.73%) and sweating (7.03%). Skin symptoms were present in 32.43% of individuals, where 1.89% had spots on the body and 5.68% reported itching.

At least one symptom related to the musculoskeletal system was reported by 19.73% of participants, including fatigue (32.16%) and joint problems such as pain or discomfort (16.22%). Regarding the symptoms associated with circulation, 5.41% reported oedema in some part of the body.

Regarding psychological symptoms, these were present in 28.38% of the participants, divided between those who felt depressed (17.30%) and those who felt some other type of anxiety (23.51%).

Finally, among the most prevalent persistent symptoms was memory loss, affecting 42.70% of participants, followed by fatigue (32.16%), anxiety (23.51%), hair loss, and dyspnoea (19.73%).

## 4. Discussion

Long COVID rates in Brazil are significant and still require investigation. The present study is innovative by extending analysis to 18 months after symptom onset, while most studies investigated shorter periods, usually between 1 and 12 months [29].

Although the emergency period has ended, knowing the portion of the population that suffers from COVID-19 sequelae and its characteristics is of paramount importance to plan care and organize health services in the long term. Our data show that neurological, respiratory, and musculoskeletal symptoms were the most common during acute SARS-CoV-2 infection. These findings align with previous systematic reviews and meta-analyses [30,31]. Concerning long COVID after 18 months of primary SARS-CoV-2 infection, the study identified a high prevalence (66.2%). Neurological, skin, and digestive disorders were the most frequent symptom clusters reported 18 months after the positive test. Studies differ in the proportion of individuals with persistent signs and symptoms, in individual characteristics, in previous health conditions (such as the acute phase of the disease), the place of treatment, and the follow-up time [7,17]. A study conducted in India, with non-hospitalized adult patients, found that 78.7% of participants reported persistence of at least one symptom 18 months after the acute onset of COVID-19 [32]. In another study, conducted with patients hospitalized in Italy, post-COVID symptoms persisted in up to 60% of patients with a mean follow-up of 17 months [33]. In Israel, patients who required hospitalization were evaluated 3 and 18 months after diagnosis, and 95.8% and 38.5%, respectively, revealed persistent symptoms [34]. In our study, the differences in prevalence may be associated with the homogeneous distribution of the sample in relation to the place of treatment of COVID-19, including outpatient (36.7%), ward (31%), and ICU (32.1%). While most researchers evaluated mild or severe cases, few of them addressed mixed populations. These variations underscore the need for standardized and validated COVID-19 research instruments to harmonize data collection, enhance quality, and diminish reporting variability.

Another finding is the association of long COVID with females (73.9%), which is agreed by the literature. A study performed in Milan (Italy), found that 69% of participants had long COVID, of which 81.7% were female, and that females were three times more likely to have persistent symptoms [18]. Another study that followed patients for six months in northwestern Spain also showed higher rates of post-COVID sequelae in females (59%) [6]. There are still no clear explanations about this phenomenon, but hormonal issues and the perception of physical and psychological health conditions are believed to be better observed in the female sex [18]. More research will be needed to better elucidate the relationship between long COVID and females.

Some research is investigating the prevalence and symptoms of long COVID in minority and disadvantaged populations. As a result, individuals with greater socioeconomic vulnerability have higher rates of mortality and severity of the disease [35,36]. Minorities and lower income populations face difficulties in accessing quality medical and hospital services, especially in countries where there are no government policies that offer such health services.

In the current study, long COVID was associated with the sedentary behaviour of the participants, corroborating other studies in the literature [37,38,39]. However, this finding should be interpreted with caution because physical inactivity is often correlated with a higher prevalence of chronic diseases, which could independently increase the risk of long COVID. Evidence also suggests that long COVID patients, especially in more severe cases, may experience post-exertional malaise or exertional intolerance (particularly those that meet criteria for myalgic encephalomyelitis/chronic fatigue syndrome), which impacts their functional capacity [40,41]. In such cases, these individuals should be monitored by a qualified professional to ensure safe physical activity [40,42].

The study indicates that poor sleep quality is associated with long COVID, in agreement with published research supporting this association [7]. A Russian study that reviewed several primary studies and reviews found that sleep disorders are related to the post-COVID period. This aggravates the health conditions of the population, since good quality sleep is fundamental for the body’s homeostasis and strengthening the immune system, as well as the functioning of the neurological and the cognitive system [43]. Therefore, special attention should be directed to patients suffering from sleep changes, as these promote higher levels of depression and anxiety, a worse quality of life, and less physical activity [44].

Although the presence of comorbidity was not significant in this sample (*p*-value = 0.06), this condition has been reported in numerous studies on COVID-19 since the beginning of the pandemic [7,15]. Patients with chronic diseases were classified as a risk group, since there was a higher frequency of severe forms in these individuals and a higher risk of mortality. The factors involved in this process are still being analyzed and described, but angiotensin-2 converting enzyme (ACE-2) is known to play an important role in the inflammatory cascade of COVID-19 and is in greater quantity in the presence of comorbidities. Therefore, the sequelae in this group may be more pronounced and last longer [45]. In the UK, a survey of 384,137 individuals diagnosed with COVID-19 identified, in a follow-up after 12 weeks, that the presence of comorbidities increased the chances of long COVID. Chronic obstructive pulmonary disease, benign prostatic hyperplasia, migraine, multiple sclerosis, and a high body mass index presented the highest associations [35]. While ACE-2 is indeed implicated in the pathophysiology of SARS-CoV-2 infection, there is currently insufficient evidence to directly link increased ACE-2 expression in individuals with comorbidities to the prolonged sequelae of long COVID.

The study showed that individuals who needed health care in the last six months, that is, one year after the COVID-19 diagnosis, were associated with long COVID. Similarly, a Danish survey compared 8983 patients who tested positive for COVID-19 but were not hospitalized with another 80,894 who tested negative. Among the positive patients, during the six-month follow-up period, 73% had a visit to the general practitioner, or were seen in an outpatient clinic or admitted to the hospital. The greater number of visits to health services, compared to those who did not have COVID-19, may be related to the presence of persistent symptoms [46]. The same study also analyzed hospitalized patients, although with a smaller sample (1310). Those who presented comorbidities and used long-term medication had a statistically higher risk of starting new drugs, treatments, and receiving other diagnoses [46].

Some researchers have already highlighted the association of moderate and severe forms of COVID-19 with chronic diseases, continuous drug use, and the place of treatment in the acute phase of the disease [7,35,36]. In this study, for example, the more severe cases led to prolonged sequelae for at least 18 months after the onset of symptoms. Similarly, a systematic review that evaluated 20 studies and 13,340 participants confirmed that individuals with severe conditions during hospitalization, who needed intensive care, were more likely to remain symptomatic after recovery [47]. Additionally, patients who required professional care and ventilatory support in the acute phase of the disease were associated with long COVID. Other studies have explored the need for the use of oxygen therapy with the persistence of symptoms of COVID-19, mainly related to the respiratory system [36,47].

As for the signs and symptoms of long COVID, several studies have focused on elucidating what they are and their prevalence in the population. However, there is a consensus that there are a variety of symptoms that affect multiple organ systems. The research found the most prevalent symptoms to be memory loss (42.7%), fatigue (32.1%), anxiety (23.5%), and dyspnoea and hair loss (19.7%). Such symptoms were associated with SARS-CoV-2 infection and were well-described in both hospitalized and non-hospitalized patients in previous studies [46]. Currently more than 200 persistent symptoms are reported after SARS-CoV-2 [6] infection. Among the most common are fatigue, memory loss, dizziness, loss of taste or smell, depression, and anxiety [15]. Fatigue stands out in reports on long COVID; some studies already analyze only this condition in the post-COVID period and relate it to the severity of the disease at the beginning of symptoms [33]. Corroborating these findings, researchers conducted an investigation in India with 200 patients, 12 weeks after primary infection. It found that 23.5% of these patients experienced fatigue, 12.5% experienced hair loss, 9% experienced dyspnoea, 4.5% experienced memory loss, and 3.5% experienced headache [48]. Another study also analyzed the same period as the present study, 18 months after infection, and identified dyspnoea in 15.8%, fatigue in 21.2%, and mental confusion in 7.3% of the participants [34]. In the United States, 16,000 Americans were evaluated two months after symptoms began, and the results were similar to the present study. Of these, 52.2% had fatigue, 39.7% reported dyspnoea, 45.7% had memory loss, and 43.7% reported a loss of smell. They also highlighted that complete vaccination for COVID-19 can be a protective factor against long COVID [29]. Prospective studies with longer follow-ups are still needed to identify the persistence of signs and symptoms in the population and the effect of vaccines on post-COVID.

The present study has some limitations that should be considered. Firstly, as in any interview, there is a potential for recall bias, as individuals may forget or omit significant details from previous events. To mitigate this bias, standardized forms and instructions for each moment of the interview were used. Secondly, the study depended exclusively on self-reported data and lacks verification of the disease by the treating physician, as well as evidence through electronic health records. The diagnosis of long COVID is based on self-reported symptoms and interview data, suggesting that a more thorough collection of experiences might more accurately represent the real-world manifestation of long COVID. To improve the precision and dependability of results, subsequent research should integrate clinical evaluations or medical documentation to accurately record long COVID symptoms and reduce subjective bias. Additionally, the cross-sectional design employed in the study hinders the ability to make definitive causal inferences. Thirdly, participants who were more accessible through telephone or digital means may have been preferentially included, potentially excluding individuals with limited technological access. Fourthly, all participants were initially identified with COVID-19 before June 2021, during which either the wild-type or alpha variant predominated and before the extensive distribution of COVID-19 vaccines [27]. This sample’s shortcomings reduce the generalizability of findings when applied to more current variants. Fifthly, there is a possibility that participants experienced asymptomatic reinfections during the study period, which were not reported or accounted for, and which may confound the results. Another limitation refers to the appearance of symptoms that may not be related to infection by SARS-CoV-2. Therefore, a comparison group would be desirable, but in the consulted databases there was no information about individuals without the disease. On the other hand, some hallmark symptoms of long COVID, such as exercise intolerance (post-exertional malaise), were not evaluated in this study. Thus, further research should aim to provide a better understanding of the risks and associations concerning chronic fatigue, quality of life and limited physical activity [49]. However, the current research contributes with new evidence about the prevalence, the main symptoms, and the factors associated with long COVID, providing information for the planning of strategies to improve the health conditions of the affected population.

## 5. Conclusions

A high prevalence of long COVID was identified in the adult population 18 months after primary SARS-CoV-2 infection. The most prevalent persistent symptoms were memory loss, fatigue, anxiety, dyspnoea, and hair loss. The proportion of individuals with long COVID was statistically higher among females, those with a family income below two minimum wages, those who were physically inactive, those who reported poor sleep quality, those who used long-term medication, those who required health care in the past six months, those who needed professional or family support, those who underwent ICU treatment, or those who used ventilatory support. Given the complexity of long COVID symptoms, significant public health resources will be required to address this condition. Longer follow-up periods will be essential to monitor these complications and generate the scientific evidence needed to support both science and society.

## Figures and Tables

**Table 1:** Sociodemographic and health characteristics of participants (N = 370).

	Long COVID			
Variables	Total ^a^	Yes (*n* = 245) ^a^	No (*n* = 125) ^a^	*p*-Value ^b^
Overall prevalence	370 (100%)	245 (66.2%)	125 (33.8%)	-
Sex				
Male	209 (56.49%)	126 (60.29%)	83 (39.71%)	**0.01**
Female	161 (43.51%)	119 (73.91%)	42 (26.09%)	
Age (years)	51 (31, 56)	50 (30, 54)	47 (28, 51)	0.29
Age group (years)				
18–30	60 (16.22%)	35 (58.33%)	25 (41.67%)	0.17
31–45	136 (36.76%)	87 (63.97%)	49 (36.03%)	
45–59	174 (47.02%)	123 (70.69%)	51 (29.31%)	
Race/ethnicity				
White	156 (42.16%)	109 (69.87%)	47 (30.13%)	0.17
Non-white	69 (18.65%)	55 (79.71%)	14 (20.29%)	
Missing data	145 (39.19%)	81 (55.86%)	64 (44.14%)	
Years of study	10 (7, 14)	9 (7, 11)	11 (8, 13)	0.36
Years of study (groups)				
<8 years of study	28 (7.57%)	23 (82.14%)	5 (17.86%)	0.28
≥8 years of study	191 (51.62%)	134 (70.16%)	57 (29.84%)	
Missing data	151 (40.81%)	88 (58.28%)	63 (41.72%)	
Married or with a partner				
No	74 (20.00%)	52 (70.27%)	22 (29.73%)	0.97
Yes	204 (55.14%)	141 (69.12%)	63 (30.88%)	
Missing data	92 (24.86%)	52 (56.52%)	40 (43.48%)	
Lives alone				
No	283 (76.49%)	190 (67.14%)	93 (32.86%)	0.55
Yes	31 (8.38%)	23 (74.19%)	8 (25.81%)	
Missing data	56 (15.13%)	32 (57.14%)	24 (42.86%)	
Family income (minimum wage ¥)				
<2	38 (10.27%)	36 (94.74%)	2 (5.26%)	**0.03**
≥2	150 (40.54%)	117 (78.00%)	33 (22.00%)	
Missing data	182 (49.19%)	92 (50.55%)	90 (49.45%)	
Smoking habit				
No	167 (45.13%)	127 (76.05%)	40 (23.95%)	1.00
Yes	63 (17.03%)	48 (76.19%)	15 (23.81%)	
Missing data	140 (37.84%)	70 (50.00%)	70 (50.00%)	
Physical activity				
No	54 (14.59%)	45 (83.33%)	9 (16.67%)	**0.02**
Yes	220 (59.46%)	145 (65.91%)	75 (34.09%)	
Missing data	96 (25.95%)	55 (57.29%)	41 (42.71%)	
Sleep pattern				
Poor	75 (20.27%)	70 (93.33%)	5 (6.67%)	**<0.001**
Good	240 (64.87%)	153 (63.75%)	87 (36.25%)	
Missing data	55 (14.86%)	22 (40.00%)	33 (60.00%)	
Comorbidity				
No	204 (55.14%)	126 (61.76%)	78 (38.24%)	0.06
Yes	166 (44.86%)	119 (71.69%)	47 (28.31%)	
Long-term medication				
No	178 (48.11%)	111 (62.36%)	67 (37.64%)	**<0.001**
Yes	78 (21.08%)	67 (85.90%)	11 (14.10%)	
Missing data	114 (30.81%)	67 (58.77%)	47 (41.23%)	
Need of social or family support				
No	183 (49.46%)	110 (60.11%)	73 (39.89%)	**<0.001**
Yes	87 (23.51%)	69 (79.31%)	18 (20.69%)	
Missing data	100 (27.03%)	66 (66.00%)	34 (34.00%)	
Place of treatment				
Ambulatory	136 (36.76%)	75 (55.15%)	61 (44.85%)	**<0.001**
Inpatient ward	115 (31.08%)	76 (66.09%)	39 (33.91%)	
ICU §	119 (32.16%)	94 (78.99%)	25 (21.01%)	
Ventilatory Support				
No	167 (45.14%)	95 (56.89%)	72 (43.11%)	**<0.001**
Yes	160 (43.24%)	124 (77.50%)	36 (22.50%)	
Missing data	43 (11.62%)	26 (60.47%)	17 (39.53%)	
Need of healthcare support in last 6 months				
No	151 (40.81%)	82 (54.30%)	69 (45.70%)	**<0.001**
Yes	142 (38.38%)	124 (87.32%)	18 (12.68%)	
Missing data	77 (20.81%)	39 (50.65%)	38 (49.35%)	
SUS †				
No	220 (59.46%)	137 (62.27%)	83 (37.73%)	0.07
Yes	150 (40.54%)	108 (72.00%)	42 (28.00%)	

¥: In 2021, the minimum wage in Brazil was R$1100.00; § ICU: Intensive Care Unit; † SUS: Unified Health System; a: Median (IQR); n (%). b: Wilcoxon rank sum test; Pearson’s Chi-squared test; Fisher’s exact test. “Missing data” were not considered in the associations. Bold means *p* < 0.05.

**Table 2:** Clinical manifestations of acute COVID-19 infection and long COVID (at the 18 month follow-up) according to the affected main organ systems.

Symptoms Clusters	Acute COVID-19 Infection (Baseline Data)		Long COVID (at the 18 Month Follow-Up)	
	Frequency ¥	Percentage (%)	Frequency	Percentage (%)
Neurological symptoms				
No	49	13.35	235	63.51
Yes	318	86.65	135	36.49
Respiratory symptoms				
No	66	17.98	261	70.54
Yes	301	82.02	109	29.46
Digestive symptoms				
No	183	49.86	257	69.46
Yes	184	50.14	113	30.54
Endocrine symptoms				
No	263	71.66	324	87.57
Yes	104	28.34	46	12.43
Skin symptoms				
No	342	93.19	250	67.57
Yes	25	6.81	120	32.43
Musculoskeletal symptoms				
No	126	34.33	297	80.27
Yes	241	65.67	73	19.73
Circulatory symptoms				
No	336	95.45	350	94.59
Yes	16	4.55	20	5.41
Psychological symptoms				
No	229	62.40	265	71.62
Yes	138	37.60	105	28.38

Neurological Symptoms (e.g., headache, change of vision, tingling or numbness in any part of the body, dizziness, loss of coordination of movements, and memory loss). Respiratory Symptoms (e.g., runny nose, sore throat, hoarse voice, cough, phlegm production, and shortness of breath). Digestive Symptoms (e.g., change in appetite, nausea, vomiting, abdominal cramps/pain, and change in stool). Endocrine Symptoms (e.g., loss of hair and perspiration). Skin Symptoms (e.g., stains on the body and body itching/itching). Musculoskeletal Symptoms (e.g., tiredness/fatigue and joint problems (such as pain or discomfort)). Circulatory Symptoms (e.g., oedema (swelling) in some part of the body). Psychological Symptoms (e.g., depressed (sad, discouraged), anxious (restless, distressed, agonized and impatient). ¥: Two missing cases.

## Data Availability

The raw data supporting the conclusions of this article will be made available by the authors on request.

## Supplementary Materials

The following supporting information can be downloaded at: www.mdpi.com/article/10.3390/healthcare13030228/s1, Table S1: Signs and symptoms in acute phase of COVID-19; Table S2: Absolute and relative frequencies of signs and symptoms of Long COVID in each organ system.
