# Supplementary material for: Self-Reported Post-COVID Symptoms at 18 Months After Infection Among Adults in Southern Brazil: A Cross-Sectional Study
Source: Healthcare (Basel). 2025 Jan 23;13(3):228. doi: 10.3390/healthcare13030228 (PMC11816678; doi:10.3390/healthcare13030228)
Supplement: Supplementary file 1 [file healthcare-13-00228-s001.zip › healthcare-3387978-supplementary.pdf]

Supplementary Table S1 – Signs and symptoms in acute phase of COVID-19

Symptoms in the Acute Phase	Frequency	Percentage
**Neurological symptoms**		
No	49	13.35%
Yes	318	86.65%
Headache		
No	169	47.08%
Yes	190	52.92%
Eye pain		
No	280	77.99%
Yes	79	22.01%
Change in vision		
No	342	95.00%
Yes	18	5.00%
Change in smell		
No	179	49.86%
Yes	180	50.14%
Change in taste		
No	184	51.25%
Yes	175	48.75%
Change in speech		
No	332	92.74%
Yes	26	7.26%
Change in hearing		
No	353	99.16%
Yes	3	0.84%
Ringing in the ear		
No	331	93.77%
Yes	22	6.23%
Tingling or numbness in any part of the body		
No	323	90.99%
Yes	32	9.01%
Dizziness		
No	272	76.62%
Yes	83	23.38%
Loss of coordination of movements		
No	324	90.50%
Yes	34	9.50%
Memory loss		
No	304	85.15%
Yes	53	14.85%
**Respiratory Symptoms**		
No	66	17.98%
Yes	301	82.02%
Runny nose		
No	265	73.61%

Yes	95	26.39%
Sore throat		
No	235	66.76%
Yes	117	33.24%
Hoarse voice (hoarseness)		
No	300	85.71%
Yes	50	14.29%
Cough		
No	162	46.42%
Yes	187	53.58%
Phlegm production		
No	294	83.52%
Yes	58	16.48%
Chest pain		
No	259	73.16%
Yes	95	26.84%
Shortness of breathe		
No	128	35.96%
Yes	228	64.04%
**Digestive Symptoms**		
No	183	49.86%
Yes	184	50.14%
Change in appetite		
No	223	63.35%
Yes	129	36.65%
Nausea		
No	282	79.89%
Yes	71	20.11%
Vomiting		
No	316	89.77%
Yes	36	10.23%
Abdominal cramps/pain		
No	307	86.97%
Yes	46	13.03%
Change in stool		
No	251	71.71%
Yes	99	28.29%
**Endocrine Symptoms**		
No	263	71.66%
Yes	104	28.34%
Loss of hair		
No	312	87.64%
Yes	44	12.36%
Perspiration		
No	279	78.81%
Yes	75	21.19%
**Skin symptoms**		
No	342	93.19%

Yes	25	6.81%
Stains on the body		
No	345	96.37%
Yes	13	3.63%
Body itching/itching		
No	344	96.63%
Yes	12	3.37%
**Musculoskeletal symptoms**		
No	126	34.33%
Yes	241	65.67%
Tiredness/fatigue		
No	125	34.92%
Yes	233	65.08%
Joint problems (such as pain or discomfort)		
No	301	85.03%
Yes	53	14.97%
**Circulatory symptoms**		
No	336	95.45%
Yes	16	4.55%
Edema (swelling) in some part of the body		
No	336	95.45%
Yes	16	4.55%
**Psychological symptoms**		
No	229	62.40%
Yes	138	37.60%
Depressed (sad; discouraged)		
No	250	70.03%
Yes	107	29.97%
Anxious (restless; distressed; agonized; impatient)		
No	243	68.84%
Yes	110	31.16%

Supplementary Table S2 – Absolute and relative frequencies of signs and symptoms of Long COVID in each organ system.

Variable	Frequency	Percentage
**Neurological symptoms – Long COVID**		
No	235	63.51
Yes	135	36.49
Headache		
No	320	86.49
Yes	50	13.51
Eye pain		
No	341	92.16
Yes	29	7.84
Change in vision		
No	300	81.08
Yes	70	18.92
Change in smell		
No	336	90.81
Yes	34	9.19
Change in taste		
No	346	93.51
Yes	24	6.49
Change in speech		
No	346	93.51
Yes	24	6.49
Change in hearing		
No	349	94.32
Yes	21	5.68
Ringing in the ear		
No	340	91.89
Yes	30	8.11
Tingling or numbness in any part of the body		
No	323	87.30
Yes	47	12.70
Dizziness		
No	335	90.54
Yes	35	9.46
Loss of coordination of movements		
No	331	89.46
Yes	39	10.54
Memory loss		
No	212	57.30
Yes	158	42.70
**Respiratory symptoms – Long COVID**		

No	261	70.54
Yes	109	29.46
Runny nose		
No	346	93.51
Yes	24	6.49
Sore throat		
No	334	90.27
Yes	36	9.73
Hoarse voice (hoarseness)		
No	339	91.62
Yes	31	8.38
Cough		
No	330	89.19
Yes	40	10.81
Phlegm production		
No	341	92.16
Yes	29	7.84
Chest pain		
No	336	90.81
Yes	34	9.19
Shortness of breathe		
No	297	80.27
Yes	73	19.73
**Digestive symptoms – Long COVID**		
No	257	69.46
Yes	113	30.54
Change in appetite		
No	332	89.73
Yes	38	10.27
Nausea		
No	354	95.68
Yes	16	4.32
Vomiting		
No	361	97.57
Yes	9	2.43
Abdominal cramps/pain		
No	353	95.41
Yes	17	4.59
Change in stool		
No	352	95.14
Yes	18	4.86
**Endocrine symptoms – Long COVID**		
No	324	87.57
Yes	46	12.43
Loss of hair		

No	297	80.27
Yes	73	19.73
Perspiration		
No	344	92.97
Yes	26	7.03
**Skin symptoms – Long COVID**		
No	250	67.57
Yes	120	32.43
Stains on the body		
No	363	98.11
Yes	7	1.89
Body itching/itching		
No	349	94.32
Yes	21	5.68
**Musculoskeletal symptoms – Long COVID**		
No	297	80.27
Yes	73	19.73
Tiredness/fatigue		
No	251	67.84
Yes	119	32.16
Joint problems (such as pain or discomfort)		
No	310	83.78
Yes	60	16.22
**Circulatory symptoms – Long COVID**		
No	350	94.59
Yes	20	5.41
Edema (swelling) in some part of the body		
No	350	94.59
Yes	20	5.41
**Psychological symptoms – Long COVID**		
No	265	71.62
Yes	105	28.38
Depressed (sad; discouraged)		
No	306	82.70
Yes	64	17.30
Anxious (restless; distressed; agonized; impatient)		
No	283	76.49
Yes	87	23.51
